# Host Cytoskeleton Gene Expression Is Correlated with the Formation of Ascovirus Reproductive Viral Vesicles

**DOI:** 10.3390/v14071444

**Published:** 2022-06-30

**Authors:** Heba A. H. Zaghloul, Peter Arensburger, Brian A. Federici

**Affiliations:** 1 Department of Botany and Microbiology, Faculty of Science, Alexandria University, Alexandria 21511, Egypt; 2 Department of Biological Sciences, California State Polytechnic University, Pomona, 3801 West Temple Avenue, Pomona, CA 91768, USA; parensburger@cpp.edu; 3 Interdepartmental Graduate Program in Microbiology, Institute for Integrative Genome Biology, University of California, Riverside, CA 92521, USA; 4 Department of Entomology, University of California, Riverside, CA 92521, USA

**Keywords:** ascovirus, cytoskeleton, mitochondria, RNA-seq, vesicles, *Trichoplusia ni*, apoptosis, dynein, actin, tubulin

## Abstract

Ascoviruses are large DNA viruses that primarily infect lepidopteran larvae. They differ markedly from other plant or animal viruses by initiating replication in the nucleus, then inducing nuclear lysis followed by extensive cellular hypertrophy and subsequent cleavage of the entire enlarged cell into numerous viral vesicles. Most progeny virions are assembled in these vesicles as they circulate in the hemolymph. Here, we report transcriptome studies of host cytoskeletal genes in larvae infected with ascoviruses from 6 h to 21 days post-infection (dpi). We focused on the cabbage looper, *Trichoplusia ni*, infected with the *Trichoplusia ni* ascovirus (TnAV), along with supporting studies on the fall armyworm, *Spodoptera frugiperda*, infected with the *Spodoptera frugiperda* ascovirus (SfAV). In *T. ni*, many cytoskeleton genes were upregulated at 48 hours post-infection (hpi), including 29 tubulins, 21 actins, 21 dyneins, and 13 kinesins. Mitochondrial genes were upregulated as much as two-fold at 48 hpi and were expressed at levels comparable to controls in both *T. ni* and *S. frugiperda*, even after 21 dpi, when several cytoskeleton genes remained upregulated. Our studies suggest a temporal correlation between increases in the expression of certain host cytoskeletal genes and viral vesicle formation. However, these results need confirmation through functional genetic studies of proteins encoded by these genes.

## 1. Introduction

Ascoviruses belong to a family of large, enveloped dsDNA viruses (Family Ascoviridae) discovered in the late 1970s [1,2]. They are known primarily from lepidopteran larvae, primarily species of *Heliothis*, *Spodoptera*, and *Trichoplusia,* in which they cause chronic diseases, often lasting for weeks, that retard growth and development, and are ultimately fatal. Ascovirus virions are large (100–400 nm), enveloped, bacilliform or reniform, and have dsDNA circular genomes ranging from approximately 150 to 190 kbp, coding for more than a hundred genes. Phylogenetically, ascoviruses are nucleocytoplasmic large DNA viruses (NCLDVs), which include viruses such as iridoviruses, phycodnaviruses, and poxviruses. These viruses may begin replication in the nucleus, but typically replicate in the cytoplasm where most DNA synthesis occurs and progeny virions form. Molecular phylogenetic studies indicate that ascoviruses evolved from iridoviruses that attack lepidopteran larvae [3,4]. Like iridoviruses, ascoviruses may pass through the midgut epithelium, but, if replication occurs there, it is minimal [5]. Thus, even if fed large doses of virions, infection rates are low [6]. Instead, in nature, ascoviruses are mechanically transmitted among lepidopteran hosts on the ovipositor of female parasitoid wasps when they lay eggs in larvae [7,8,9]. This is easily mimicked in the laboratory using fine pins dipped in a suspension of virions [10].

Whereas the unique characteristics of virion structure, gross pathology, and genomics easily differentiate ascoviruses from all other viruses, by far their most novel features are the extraordinary changes they induce in the architecture of infected cells for reproduction [11,12]. Within a few hours of infection, the initiation of replication causes the nucleus to rupture as the cell begins to enlarge. This process resembles apoptosis, and, in fact, the *Spodoptera frugiperda* ascovirus (SfAV-1a), the type species, synthesizes an executioner caspase that degrades host DNA at this stage [13,14,15]. Subsequently, the anucleate cell enlarges at least five to ten times or more, during which the mitochondria multiply and move to cleavage planes throughout the mixture of nucleoplasm and cytoplasm [2,11,16]. As the cytopathology progresses, masses of membrane are formed de novo between the arrays of mitochondria. Concomitantly, the plasmalemma, assisted by mitochondria, invaginates to connect to the planes of membrane. At about this stage, low numbers of progeny virions are observed. The membrane being synthesized becomes the limiting membrane of numerous viral vesicles the virus now cleaves from the cell. These nascent viral vesicles spill into the larval hemolymph, where most DNA replication and progeny virion assembly occurs.

Ascovirus research is a young and small field in which most studies have focused on finding and characterizing new isolates, along with fundamental studies on their genomics, phylogenetics and transmission, and initial basic studies on ascoviruses in lepidopteran cell lines [13,17,18,19,20]. The latter studies have provided interesting new information, but they reflect poorly the formidable changes in cells, especially the massive production of viral vesicles that occur in larvae. To date, ascovirus cytopathology has been largely descriptive, and has been based mostly on light and electron microscopy. However, it is well known that several other insect viruses manipulate host cytoskeleton proteins, especially actin, and motor proteins for virus reproduction [21]. The main current limitation to conducting studies on ascoviruses is the lack of a suitable in vitro system where the cytopathological changes observed in vivo can be reproduced [17]. Thus, in the present study, we examined the effects of the *Trichoplusia ni* ascovirus (TnAV) on the host cytoskeleton and motor protein gene expression, along with mitochondrial gene expression, using RNA-sequencing, sampling infected larvae from 6 h to 2 days post-infection (dpi) and again 7, 14, 21 days later. We also included similar mitochondria studies for *S. frugiperda* larvae infected with SfAV. The *T. ni* host transcriptome revealed that significant upregulation of certain cytoskeleton genes occurred at 48 hpi in a combination of tissues that included the epidermis, tracheal matrix, and fat body, which occurred prior to the initial accumulation of vesicles in the hemolymph. Upregulated cytoskeletal genes coding for proteins involved with cell architecture included 29 for tubulins, 21 for actins or actin-related proteins, and 21 and 13, respectively, for dyneins or kinesins, which are known to be involved with moving mitochondria.

## 2. Materials and Methods

### 2.1. Preparation of TnAV Viral Vesicles and Virions for Infection

The stock of TnAV-6a1 [22] viral vesicles and virions were purified according to the methods described by Bideshi et al. 2018 [15] and preserved at −80 °C until used to inject *T. ni* larvae (late 3rd or early 4th instars). Hemolymph packed with viral vesicles was collected after 9 dpi and preserved immediately at −80 °C for later use.

### 2.2. Infection of T. ni Larvae with Viral Vesicles

*T. ni* 3rd instar larvae obtained from Benzon Research, Carlisle, PA were raised at room temperature (22 °C) and fed a general noctuid artificial diet on a regular basis. Third instars were infected with the −80 °C preserved hemolymph packed with TnAV viral vesicles by piercing them through the cuticle with a minutin pin dipped in a suspension of 10^8^ vesicles/mL determined with a hemocytometer. Control larvae were mock-infected with a phosphate-buffered saline (PBS). Infected larvae were observed for three weeks post-infection to collect different body tissues for subsequent transcriptomic analysis. The presence of a milky white hemolymph, characteristic of viral vesicles, was confirmed visually for all RNA-sequencing samples starting from 48 hpi and up to 21 dpi samples.

### 2.3. Light and Electron Microscopy

For light and electron microscopy, tissues were fixed in 3% glutaraldehyde and 1% OsO_4_ (osmium tetroxide), dehydrated in a progressive ethanol series, and embedded in Epon-Araldite using methods described previously [1].

### 2.4. RNA Isolation and Sequencing

The total RNA was collected from TnAV-infected *T. ni* hemolymph or body tissues at early and late time-points. Specifically, at early time-points (6–48 h post-infection) hpi, we collected the hemolymph or fat body, epidermis and tracheal matrix combined. Similarly, at late time-points (7, 14, and 21 days post-infection) dpi, we collected the hemolymph or fat body, epidermis, tracheal matrix and alimentary canal combined. In the case of hemolymph, a 100 µL sample was collected at 0, 6, 12, 24, 48 hpi. Furthermore, the same hemolymph amount was collected after 7, 14 and 21 days post-infection. In the case of body tissues (referred to as somatic tissues), the sample collection was carried out in parallel at both early and late time-points. For early time-points, samples of fat body, tracheal matrix and epidermis were collected at the above-mentioned hours from TnAV-injected *T. ni*. For late time-points, samples of the fat body, tracheal matrix, epidermis and alimentary canal were collected on the above-mentioned days. PBS, rather than TnAV viral vesicles, were used for injection of control larvae. Detailed descriptions of the RNA isolation protocol and the preparation of single-end libraries for RNA-sequencing are provided in [23]. For sequencing, HiSeq2500 and NextSeq500 sequencers (Illumina) were used to sequence early and late samples, respectively, at the Core Facility, UCR Institute for Integrative Genome Biology, U.S.A. For the early-time-point samples, there were two replicates collected and sequenced, whereas for later time-points, three replicates were collected and sequenced for each tissue. For early time-points, the 6 and 12 hpi expression levels were compared to 0 h PBS-injected control larvae. The 24 hpi and 48 hpi expression levels were compared to 24 and 48 h controls, respectively. For late time-points, the 7, 14 and 21 dpi samples were compared to 0 h PBS-injected control larvae which served as control samples.

### 2.5. Analysis of Cytoskeleton and Mitochondrial Gene Expression in Hemolymph Viral Vesicles versus Somatic Tissues at Early and Late Time-Points Post-Infection

To study the expression of cytoskeleton and mitochondrial genes, the *T. ni* genome (accession number: ASM360422v1) available through http://www.tnibase.org/cgi-bin/index.cgi, was searched for the following: actin, actin-related proteins, dynein, kinesin, myosin, tubulin, dynactin, laminin, lamin, profilin, cofilin and filamin. This search led to the identification of 206 cytoskeleton genes. For the *T. ni* mitochondrion (GenBank accession number MK714850.1) or the *S. frugiperda* mitochondrion (GenBank accession number KM362176), complete genomes were used to study the expression of this organelle’s genes in the above lepidopteran larvae. Gene expression levels (RPKMs) [24] were calculated using the methodology described in [23]. In brief, after mapping reads to the appropriate reference (i.e., *T. ni* genome, TnAV genome, or mitochondrial genome), we identified those reads that mapped in the same orientation as the gene ORF they mapped to, and RPKM quantification of each gene was calculated using these correctly oriented reads. The data are reported for every cytoskeleton and mitochondrial gene in Supplementary Tables S1–S4 and S5–S10, respectively. GraphPad Prism Software, San Diego, CA, USA, www.graphpad.com accessed on 29 December 2021, was used to produce heatmaps and histograms, and for the generation of pie charts. An unpaired *t*-test was used to assess the statistical significance of the differences in *T. ni* cytoskeleton gene mean expression levels between control and infected samples at each time-point post-infection.

### 2.6. Data Availability

The raw RNA-Seq data analyzed in this study can be accessed through GSE114902 and GSE174236 accession numbers for early and late time-points post-infection, respectively.

## 3. Results

### 3.1. Microscopic Examination of the TnAV Progressive Stages of Infection Development in Third and Fourth Instar Larvae of T. ni

**Figure 1:**
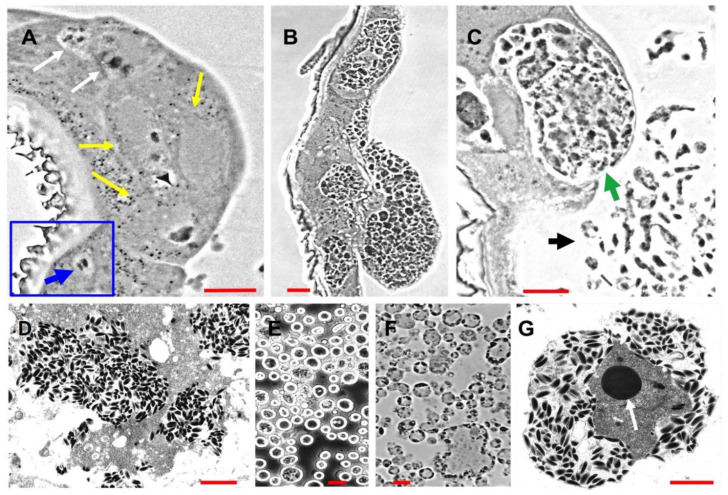
Micrographs of progressive stages of *Trichoplusia ni* ascovirus (TnAV) pathogenesis and viral vesicle formation in 3rd and 4th instars of *Trichoplusia ni*. (**A**) The two white arrows indicate, respectively, an early stage of nucleus hypertrophy and an adjacent cell in which the nucleus has lysed in the epidermis. The yellow arrows point to three cells at a later stage of hypertrophy prior to vesicle formation. The inset outlined in blue shows a typical nucleus prior to hypertrophy and lysis (blue arrow). This stage of pathogenesis is observed in clusters of cells ranging throughout somatic tissues (primarily fat body, epidermis, and tracheal matrix) from as early as six hours until at least 72 h post-infection (hpi). (**B**) Clusters of epidermal cells in which numerous viral vesicles have formed prior to their release into the hemolymph. This stage is observed more commonly at 48–72 hpi and thereafter. (**C**) A cell containing viral vesicles and cell fragments with aggregations of developing virions at about 72–96 hpi. The green arrow points to a greatly hypertrophied anucleate epidermal cell about to lyse, releasing nascent viral vesicles into the hemolymph. The black arrow points to nascent viral vesicles and cell fragments in the hemolymph from other lysed cells. (**D**) Electron micrographs of a fragmented cell containing numerous virions. (**E**,**F**) Light micrographs of, respectively, a phase contrast wet mount preparation and a plastic section through viral vesicles characteristic of the hemolymph beginning from 48 hpi–7 days post-infection (dpi) until death of the larvae as long as six weeks later. (**G**) Electron micrograph of an ultrathin section through a mature viral vesicle filled with virions outside the virogenic stroma. The dense core (arrow) in the center is likely the site of viral DNA synthesis, with the surrounding grey area being the site of viral protein synthesis and progeny virion assembly. (**A**–**C**) are phase contrast micrographs of Epon-Araldite sections. Red bars = 10 µm. Red bar in (**D**) = 1 µm, (**E**) and (**F**) = 10 µm, and (**G**) = 1 µm.

In previous studies of ascovirus pathogenesis and cytopathology in vivo, it has been conservatively estimated that the number of viral vesicles produced from each infected cell was about 20 [2,5,16]. Most of these studies were conducted with 2nd to 4th instar noctuid larvae. However, our current study, prompted by our transcriptome data, indicated that the numbers of vesicles generated per cell can be much larger (Figure 1). Estimates made from micrographs (Figure 1B,C) indicated that the number of nascent vesicles with a diameter of about 2–5 microns may have been as much as fifty or more per cell. This is a significant increase from previous estimates and a subject worthy of more detailed study, both with respect to the numbers and size per cell, and the mechanisms underlying the cleavage of cells controlled by ascoviruses.

### 3.2. Transcriptome of T. ni Cytoskeleton Genes in Somatic Tissues versus Hemolymph Viral Vesicles

**Figure 2:**
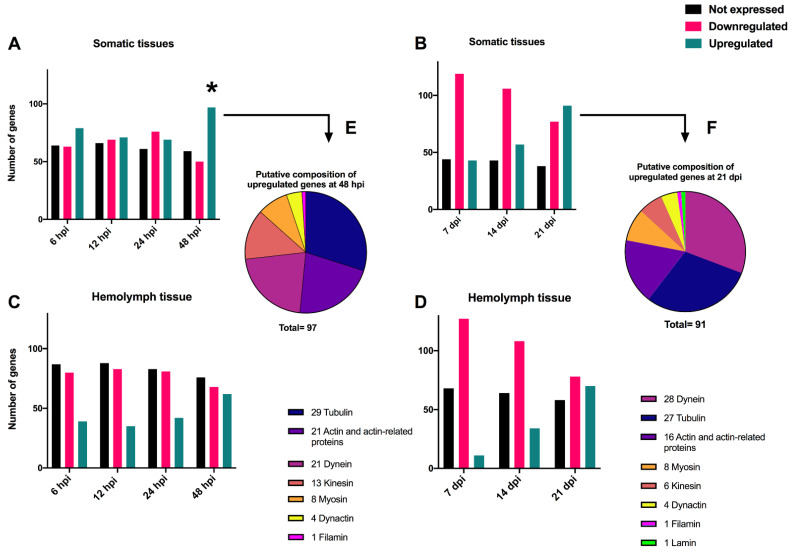
Histograms showing the number of unexpressed, upregulated and downregulated cytoskeleton *Trichoplusia ni* genes in somatic (**A**,**B**) and hemolymph (**C**,**D**) tissues after infection with TnAV. The upregulated and downregulated genes were expressed at levels greater or lower than those in control larvae. Unexpressed genes were those for which no changes were detected (0 RPKM) between infected and control larvae. In (**B**–**D**), expression levels are compared to 0 h in control larvae. In (**A**), the 6 and 12 hours post-infection (hpi) expression levels were compared to 0 h control larvae. The 24 hpi and 48 hpi expression levels were compared to 24 and 48 h controls, respectively. The putative composition of the upregulated *T. ni* genes at 48 hpi and 21 days post-infection (dpi) is illustrated by piecharts in (**E**,**F**). The * refers to the time point post-infection where a statistical significant upregulation in cytoskeleton gene expression levels was detected in comparison to controls collected in the same time point.

The transcriptomes of 206 cytoskeleton genes identified in the *T. ni* genome were examined in somatic and hemolymph tissues. The analysis of the *T. ni* somatic tissues at early time-points revealed certain cytoskeleton genes were upregulated while others were downregulated after infection with TnAV compared to the expression levels of control larvae. Specifically, 79 and 71 were upregulated at 6 and 12 hpi, respectively, and 69 and 97 at 24 and 48 hpi, respectively. Alternatively, 63 and 69 were downregulated at 6 and 12 hpi, respectively, and another 76 and 50 at 24 and 48 hpi, respectively. The level of downregulation ranged from (1- to 24.7-fold). The identity of the proteins coded for by 97 upregulated *T. ni* genes at 48 hpi were 29 tubulins, 21 actins or actin-related proteins, 21 dyneins, 13 kinesins, 8 myosins, 4 dynactins and 1 filamin (Figure 2). The level of upregulation ranged from (1- to 142-fold). Numerous other host cytoskeleton genes (59–66 genes) of the same types showed no expression (0 RPKM) before or after infection. A comparison of the log2 RPKM means of *T. ni* cytoskeleton genes at 6 and 12 hpi samples against the 0 h control by unpaired *t*-test revealed that the differences in their means were not statistically significant.

The mean expression levels of cytoskeleton genes in control larvae (24 h) were not significantly different at 24 hpi; differences in these genes were only statistically different compared to those in control (48 h) larvae at 48 hpi.

On the other hand, we did not detect statistically significant variation between the means of expression levels of cytoskeleton genes in control and post-infection at 7, 14, and 21 dpi (Figure 2, Table 1). However, computing the fold changes of cytoskeleton genes in somatic tissues demonstrated that some genes were consistently upregulated or downregulated, by more than an at-least two-fold change in one of the three tested time-points, compared to the control expression level. Interestingly, the genes that were constantly upregulated in these tissues were as follows: 10 tubulins and 8 dyneins (Table 1). On the other hand, the genes that were constantly downregulated in these tissues were as follows: 2 actin, 1 cofilin, 2 tubulin, 5 kinesin, 5 myosin, and 1 lamin (Table 1). Overall, the study of somatic tissues at early and late time-points revealed that the host cytoskeleton gene expression levels were altered at 48 hpi, or around the time vesicles develop.

Following the same host 206 cytoskeleton genes in the hemolymph tissue at early time-points showed that many genes were downregulated when compared to 0 h control expression levels. For instance, at 6, 12, 24, and 48 hpi, 80, 83, 81, and 68 genes were downregulated, respectively. The level of downregulation ranged from 1- to 3-fold. Furthermore, there were 87, 88, 83, and 76 cytoskeleton genes that were not expressed or were undetectable (0 RPKM) before or after infection at 6, 12, 24 and 48 hpi, respectively. At the same time-points, the levels of some genes increased slightly. There were 39, 35, 42 and 62 genes that were correspondingly elevated. The level of upregulation ranged from 1- to 9.5-fold. However, using an unpaired *t*-test, none of the identified variations between the log2 RPKM averages of *T. ni* cytoskeleton genes post-infection at any of the above-described periods and the 0 h control were statistically significant. Moreover, analysis of the host cytoskeleton genes in hemolymph tissue at late time-points showed that there were 127, 108 and 78 downregulated genes after 7, 14 and 21 dpi, respectively. The level of downregulation ranged from 1- to 9.5-fold. The number of unexpressed or undetectable genes (0 RPKM) ranged from 58 to 68 genes at the examined late time-points. On the other hand, there was a gradual increase in the number of upregulated genes, as there were 11, 34 and 70 upregulated genes after 7, 14 and 21 dpi, respectively. The level of upregulation ranged from 1- to 78.7-fold. The gradual increase in the number of upregulated genes in these very late stages of the infection may have reflected leakage of the cytoskeleton gene transcripts from deteriorated somatic tissues into the hemolymph or RNA derived from hemocytes in the hemolymph.

### 3.3. Transcriptome of T. ni Mitochondrial Genes in Somatic Tissues versus Viral Vesicles at Early and Later Time-Points after TnAV Infection

**Figure 3:**
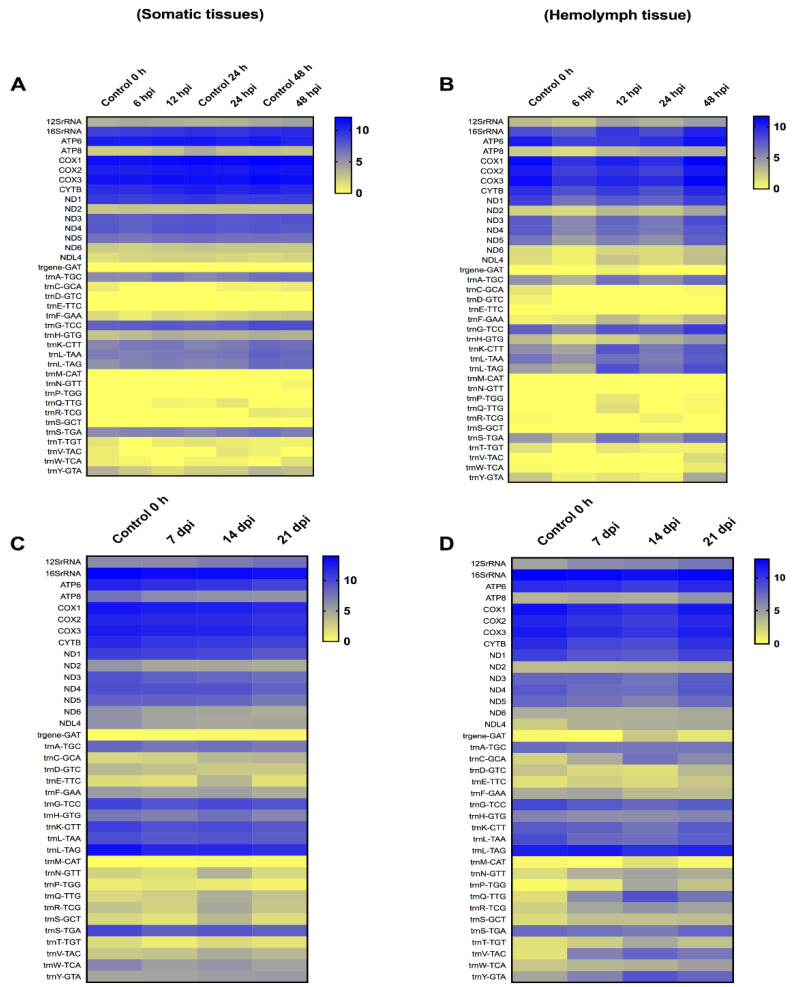
Heat-map representation of *Trichoplusia ni* temporal expression trend for mitochondrial genes after infection with *Trichoplusia ni* ascovirus (TnAV). (**A**,**C**) represents the mitochondrial transcriptome in somatic tissues in early (6–48 h post-infection, hpi) and late (7–21 days post-infection, dpi) time-points, respectively. (**B**,**D**) represents the mitochondrial transcriptome in hemolymph viral vesicles in early (6–48 hpi) and late (7–21 dpi) time-points, respectively. The color scale reflects the log2 RPKM value derived from average replicate expression levels at each represented time-point. The expression levels of control samples collected at 0–48 h (**A**) or 0 h (**B**–**D**) are depicted in each heatmap.

The *T. ni* mitochondrial transcriptome at early time-points (6–48 hpi) in somatic tissues demonstrated an immediate increase in certain mitochondrial protein coding genes at 6 hpi, followed by the upregulation of all these genes at 12 hpi. The level of upregulation ranged from 1.01- to 1.38-fold. However, at 24 and 48 hpi, most of these genes started to decrease. The level of downregulation for these genes ranged from 1.01- to 1.28-fold. The 12SrRNA and 16SrRNA were both upregulated by 1.01- to 1.09-fold, respectively, in all the early time-points, except for 16SrRNA that showed a slight decrease at 24 hpi only (1.001-fold). On the other hand, in the hemolymph tissue, all the protein-coding genes were downregulated at 6, 12 and 24 hpi, except ATP synthase F0 subunit 8 (ATP8), NADH dehydrogenase subunit 2 (ND2), and NADH dehydrogenase subunit L4 (NDL4) that started to increase constantly from 12 hpi to 48 hpi. However, at 48 hpi the 13 protein-coding genes were upregulated. The level of upregulation ranged from 1.01- to 1.90-fold. The 12SrRNA and 16SrRNA were constantly upregulated, starting at 12 hpi, and continued to increase up to 48 hpi in comparison to control levels of expression. The range of upregulation ranged from 1.01- to 1.45-fold. At 48 hpi, thirteen tRNAs were also upregulated, indicating that the mitochondrial translational machinery was functional (Figure 3).

The *T. ni* mitochondria transcriptome at late time-points (7, 14, and 21 dpi) in somatic tissues showed that the 13 protein-coding genes were downregulated. However, the level of decrease ranged from only 1.01- to 1.33-fold. The 12S rRNA showed a slight increase, starting at 14 dpi, by 1.10- to 1.20-fold. Moreover, most 9 tRNAs were downregulated, with only 6 tRNAs being upregulated. The remaining tRNAs fluctuated. In the hemolymph, only three protein-coding genes were upregulated. Specifically, upregulation ranged from 1.06- to 1.43-fold for ATP8, 1.03- to 1.17-fold for ND2, and 1.54- to 1.71-fold for NDL4. ATP synthase F0 subunit 6 (ATP6), NADH dehydrogenase subunit 3 (ND3) and NADH dehydrogenase subunit 6 (ND6) showed slight increases, but only at 21 dpi by 1.02, 1.05 and 1.04-fold, respectively. A consistent upregulation of the 12SrRNA at the three later time-points was detected, indicating the mitochondria were still active, as the upregulation ranged from 1.26- to 1.48-fold. Moreover, the transcription of 11 tRNAs was consistently increased, with upregulation ranging from 1.21- to 27.11-fold. Only five tRNAs were consistently downregulated, whereas the remaining tRNAs fluctuated.

### 3.4. Transcriptome of Spodoptera Frugiperda Mitochondrial Genes in Somatic Tissues versus Viral Vesicles in Late Time-Points

**Figure 4:**
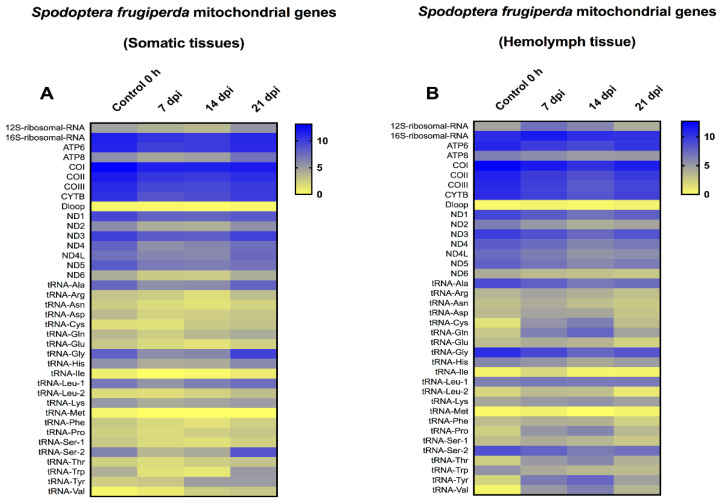
Heat-map representation of *Spodoptera frugiperda* temporal expression trend for mitochondrial genes after infection with *Spodoptera frugiperda* ascovirus (SfAV). (**A**,**B**) represents the mitochondrial transcriptome in somatic tissues and hemolymph tissue at late time-points (7–21 days post-infection, dpi), respectively. The color scale reflects the log2 RPKM value derived from average replicate expression levels at each represented time-point. The expression levels of control samples collected at 0 h are depicted in each heatmap.

In a previous study, we investigated the mitochondrial transcriptome for *S. frugiperda*, during infection with the *Spodoptera frugiperda* ascovirus (SfAV), at early time-points [25]. In that study, *S. frugiperda* larvae were not dissected, thus the mitochondrial transcriptome was examined for the entire body. As a result, in the current study, we investigated the mitochondrial transcriptome at the later time-points of 7, 14, and 21 dpi, with special emphasis on the viral vesicles fraction, i.e., the hemolymph (Figure 4). Similar to *T.ni*, mitochondrial transcriptome analysis of *S. frugiperda* somatic tissues revealed that all the mitochondrial protein-coding genes were downregulated at these late time-points. The level of decrease ranged from 1.01- to 1.56-fold. However, ATP8, NDL4 and ND6 showed a slight increase at 21 dpi by 1.24-, 1.05- and 1.01-fold, respectively. Moreover, the 12SrRNA was slightly increased in the same day by 1.13-fold, and 15 tRNAs increased in expression levels by 1.03- to 6.84-fold. However, the mitochondrial transcriptome in the hemolymph tissue revealed that all the protein-coding genes were downregulated at the later time-points. The level of decrease ranged from 1.1- to 1.60-fold when compared to the 0 h control samples. Only the 16SrRNA and 12SrRNA were upregulated at 7 or up to 14 dpi, respectively. For 12S rRNA, the level of upregulation ranged from 1.35- to 1.57-fold. Moreover, many tRNAs were upregulated at the three time-points tested. Specifically, there were 17, 15 and 10 upregulated tRNAs after 7, 14 and 21 dpi, respectively. The level of increase in tRNA expression levels ranged from 1.01- to 15.38-fold. This result implies that the *S. frugiperda* mitochondrial translation machinery was active even at these late time-points, especially in the hemolymph vesicles fraction. Furthermore, the changes in the mitochondrial 13 protein-coding genes were typically less than two-fold in comparison to control levels.

## 4. Discussion

Analysis of *T. ni* genes in somatic and hemolymph tissues after infection with TnAV revealed significant changes in the expression levels of certain cytoskeleton genes, especially those coding for structural and motor proteins (Figure 2). Upregulation of *T. ni* cytoskeleton genes in somatic tissues was only detected after 48 hpi, which is about the time viral vesicles are easily detected in the hemolymph (Figure 1). The proteins coded for by these upregulated genes were identified as tubulins, actins and mitochondrial-movement proteins, namely, dyneins and kinesins [26,27]. Although we did not detect significant upregulation of these genes before 48 hpi, this had to be occurring in at least a low percentage of somatic cells to result in the appearance of viral vesicles in the hemolymph. This suggests that more detailed studies are needed of larval histopathology early after infection.

An interesting aspect of cytoskeleton gene expression in somatic tissues at the later time-points of 7, 14, and 21 days was that several genes coding for tubulins and dyneins continued to be upregulated. These results imply that TnAV modulates the expression of certain cytoskeleton genes as the expression spreads though the body, which could assist in reshaping the large, infected cells by reorganizing the distribution of mitochondria to enhance the production of large groups of nascent vesicles. Previous transcriptomic studies conducted on another ascovirus, *Heliothis virescens* ascovirus, infecting the larvae of *Spodoptera exigua* [28], demonstrated that host genes were initially downregulated at very early time-points (6–12 hpi). Subsequently, as viral vesicle formation began to accelerate (12–72 hpi), the upregulation of certain host genes increased, including those coding for actins. Of the 206 cytoskeleton genes included in our study, we observed that many were downregulated in the hemolymph at early time-points, which was then followed by an increase in transcription during later time-points (Figure 2C,D). This increase probably did not reflect actual upregulation, but rather the leakage of these transcripts from highly deteriorated somatic tissues later in infection. Further studies are needed to confirm this hypothesis.

Another aspect worthy of note is that many host cytoskeleton genes in *S. frugiperda* infected with SfAV were also elevated starting at 48 hpi [25]. However, in that study the entire body of *S. frugiperda* larvae was triturated for RNA extraction. No separation of the vesicles fraction was performed.

Changes in the distribution of mitochondria are also known to occur in cells infected with other viruses, as described in [29]. Mitochondria were found to be altered in two ways, either by gathering near the virus factories or by cordoning these off within the cytoplasm to stop the release of apoptosis mediators. These observations were reported in the case of frog virus 3 infecting fibroblasts [30], hepatitis B virus (HBV) [31], hepatitis C virus (HCV) [32], human immunodeficiency virus-1 (HIV-1) [33] and African swine fever virus (ASFV) [34].

Our analysis of the *T. ni* mitochondrial transcriptome after TnAV infection revealed two important main characteristics of mitochondria behavior after ascovirus infection. First, this organelle was conserved (i.e., expression levels were comparable to those in the control larvae even after 21 dpi) and was metabolically active in infected cells in vivo throughout at least the three-week period of our study. During this period, additional cells were probably infected as the virus spread to uninfected cells, thereby increasing the numbers of mitochondria in the body compared to control larvae, which by that time had pupated. Second, the changes in transcription of the 13 protein-coding mitochondrial genes were not high, typically in the range of 1- to 2-fold upregulation or downregulation at any time-point tested in any tissue. In a previous study of SfAV infection of *S. frugiperda*, we followed the host mitochondrial transcriptome from 6 hpi to 7 dpi [25]. In the latter study, similar to our findings here in *T. ni*, the mitochondrial transcriptome of infected *S. frugiperda* larvae confirmed the maintenance rather than destruction of mitochondria, as occurs during apoptosis.

Another interesting feature of ascovirus infection is the upregulation of mitochondrial ribosomal activity. As shown in Figure 3 and Figure 4, the 12SrRNA and/or 16SrRNA and tRNAs were upregulated at many time-points post-infection. The preservation of this organelle and the upregulation of the mitochondrial translational machinery are consistent with human cytomegalovirus (HCMV) infection. This virus was found to upregulate both the mitochondrial transcription and translation machineries [35]. However, other viruses such as herpes simplex virus-1 (HSV-1) were found to destroy this organelle’s DNA mainly to escape its role in energy generation and antiviral activity [36]. A similar behavior was observed in HIV/HCV coinfected people, where mtDNA destruction has also been documented [37]. Further studies require to be conducted on insect viruses to define the role played by mitochondria in more detail.

Regardless of the cytopathology investigations conducted in previous studies [2,5,16] as well as the current study (Figure 1, Figure 2, Figure 3 and Figure 4), the specific molecular cytoskeleton and mitochondria events at the molecular level that result in viral vesicle formation remain unknown, and thus need to be defined by functional genetic studies, especially for proteins coded for by genes expressed in larvae infected with ascoviruses. In other viruses where this has been studied, mitochondrial maintenance and expression has been associated with mitochondrial import of many nuclear-encoded proteins [38]. In fact, one of the most important consequences of apoptosis is the loss of the mitochondrial structural and bioenergetic integrity and functions due to nuclear lysis [39], which we have shown clearly did not occur during ascovirus infections. Ascoviruses present a very different paradigm in that the nucleus and nuclear DNA are degraded after infection, yet mitochondria not only survived but proliferated and were functional. Thus, a major enigma, solved by the virus, but yet to be understood, is how mitochondrial genome replication, transcription and translation are rescued and maintained without a viable nucleus. Fragments of the nucleolus are partitioned among the cell as it cleaves, and this is where the answer to mitochondria survival may be found.

Aside from the enigma of mitochondria survival after nuclear lysis, it is impossible to over-emphasize how remarkably different the changes in cell architecture controlled by ascoviruses are compared to all other known RNA or DNA viruses. Simply put, no other known virus manipulates the host cytoskeleton nearly as much as ascoviruses. Numerous recent reviews, including [40,41,42,43], have shown how many plant and animal viruses form compartments in either the nucleus or cytoplasm where most viral genome replication and production of progeny virions occurs. As complex as these compartments are, including the interactions with host cytoskeleton proteins, they are almost trivial in comparison to the underlying molecular mechanisms that ascoviruses use to manipulate host cytoskeleton genes for viral vesicle synthesis and virion reproduction. Functional genetic studies, both in vivo and in vitro, are needed to determine the roles of the various structural proteins and enzymes that ascoviruses use to manipulate the host cytoskeleton for reproduction.

## Figures and Tables

**Table 1:** Fold changes in expression of *Trichoplusia ni* cytoskeleton genes in somatic tissues after 7, 14 and 21 days infection with TnAV compared to expression levels in mock-infected control larvae.

Genes *	Putative Function	7 dpi	14 dpi	21 dpi
Tni06G02960	Axonemal dynein light chain	2.1 **	8.9	9.2
Tni12G03140	Cytoplasmic dynein 2 light intermediate chain 1	1.5	3.6	3.9
Tni17G03100	Dynein intermediate chain 2, axonemal	18.8	69.4	89.1
Tni20G03090	Dynein light chain roadblock-type 1	1.9	5.5	6.3
Tni07G01520	Dynein, light chain, Tctex-type 1	1.4	3.7	3.6
Tni01G03980	Dynein heavy chain 6, axonemal	1.6	1.3	2.9
Tni26G01370	Dynein light chain roadblock-type	1.5	3.0	3.4
Tni15G02110	Dynein light chain 1, axonemal	1.0	2.8	3.0
Tni27G00100	Tubulin beta-2C chain	1.1	3.2	3.5
Tni04G01530	Tubulin monoglycylase TTLL3	1.2	3.6	3.7
Tni17G03250	Tubulin-tyrosine ligase	1.3	3.7	3.8
Tni17G03240	Tubulin-tyrosine ligase	2.2	7.6	9.5
Tni21G02320	Tubulin polyglutamylase TTLL13	1.3	3.6	4.0
Tni04G01780	Tubulin polyglutamylase complex subunit 2	1.5	1.2	4.4
Tni23G00310	Tubulin alpha-1A chain	1.9	2.7	2.5
Tni15G06480	Tubulin beta chain	1.1	3.0	3.1
Tni01G00970	Tubulin alpha-1 chain	1.0	2.9	3.0
Tni22G06020	Tubulin alpha chain	1.1	3.3	3.6
Tni08G04820	Actin	−1.4	−4.2	−2.5
Tni20G03470	Actin-related protein 8	−1.9	−1.9	−2.0
Tni24G01580	Cofilin/actin-depolymerizing factor-like protein	−1.7	−3.3	−1.6
Tni19G00860	Dim gamma-tubulin 3	−1.01	−2.4	−1.3
Tni23G04120	Kinesin-like protein	−1.3	−2.1	−2.2
Tni25G01890	Kinesin-like protein	−1.05	−2.9	−1.5
Tni07G03800	Kinesin-like protein	−2.2	−1.9	−2.2
Tni13G00910	Kinesin-like protein	−1.3	−3.1	−1.1
Tni05G05840	Kinesin-like protein	−1.2	−1.7	−2.2
Tni11G03970	Myosin-M heavy chain	−6.2	−5.7	−1.4
Tni02G02180	Myosin-Va	−1.03	−2.9	−1.9
Tni10G03290	Myosin heavy chain kinase D	−2.0	−13.0	−3.5
Tni00G00940	Myosin-J heavy chain	−2.0	−1.3	−1.1
Tni17G04810	Myosin-XV	−10.7	−2.8	−5.2
Tni06G04970	Nuclear lamin L1 alpha	−1.5	−3.3	−1.3
Tni31G01770	Tau-tubulin kinase 1	−2.0	−2.5	−2.7

* The *Trichoplusia ni* gene number is derived from the *T. ni* genome (accession number: ASM360422v1) available through: http://www.tnibase.org/cgi-bin/index.cgi, accessed on 22 March 2021. ** Only genes that changed by 2-fold or more, at least at one time-point post-infection, are represented in this table, and only genes that had a consistent pattern of increase or decrease at the three time-points sampled post-infection are included. Genes with a 0 expression RPKM value before or after infection are not included in this table. Days post-infection (dpi).

## Data Availability

All the data analyzed in this study can be accessed through GSE114902 and GSE174236 accession numbers.

## Supplementary Materials

The following supporting information can be downloaded at: https://www.mdpi.com/article/10.3390/v14071444/s1, Tables S1–S4: Expression levels of *Trichoplusia ni* cytoskeleton genes in somatic and hemolymph tissues after TnAV infection; Tables S5–S8: Expression levels of *Trichoplusia ni* mitochondrial genes in somatic and viral vesicles after TnAV infection at early (6–48 hpi) and late (7–21 dpi) time-points. Tables S9–S10: Expression levels of *Spodoptera frugiperda* mitochondrial genes in somatic and viral vesicles after SfAV infection at late time-points (7–21 dpi). Supplementary Figure S1: Expression levels of *Trichoplusia ni* cytoskeleton genes in somatic and hemolymph tissues after TnAV infection.
