# Supplementary figures and images for: Host Cytoskeleton Gene Expression Is Correlated with the Formation of Ascovirus Reproductive Viral Vesicles
Source: Viruses. 2022 Jun 30;14(7):1444. doi: 10.3390/v14071444 (PMC9319082; doi:10.3390/v14071444)

## Somatic tissues

**A**

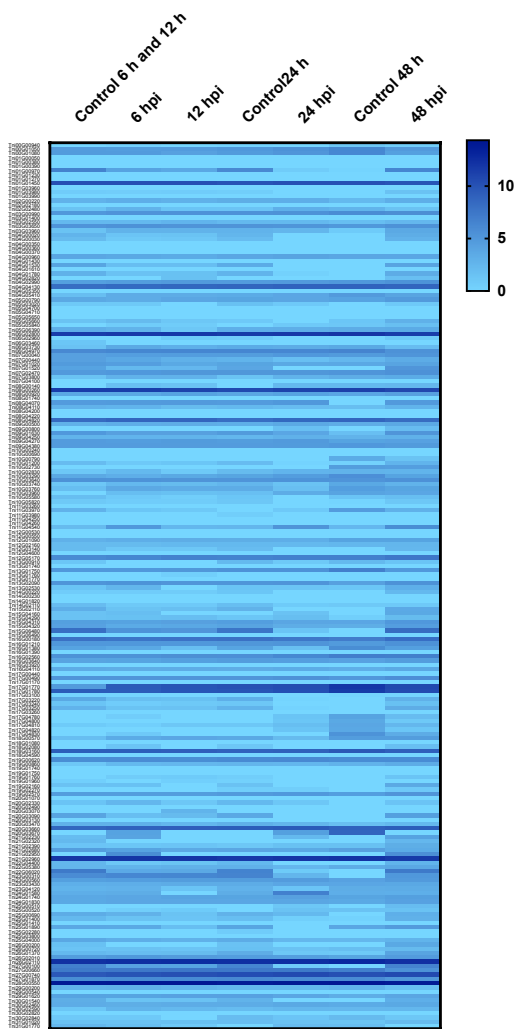

## Hemolymph tissue

**B**

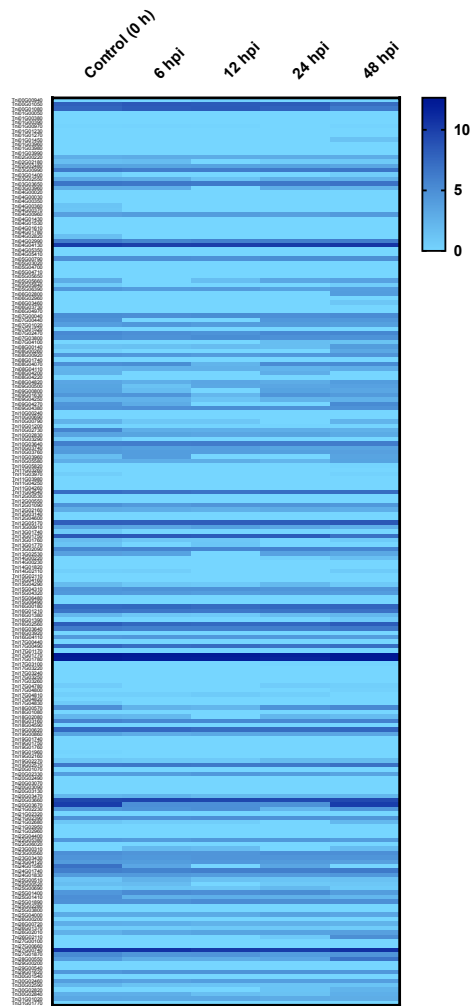

**C**

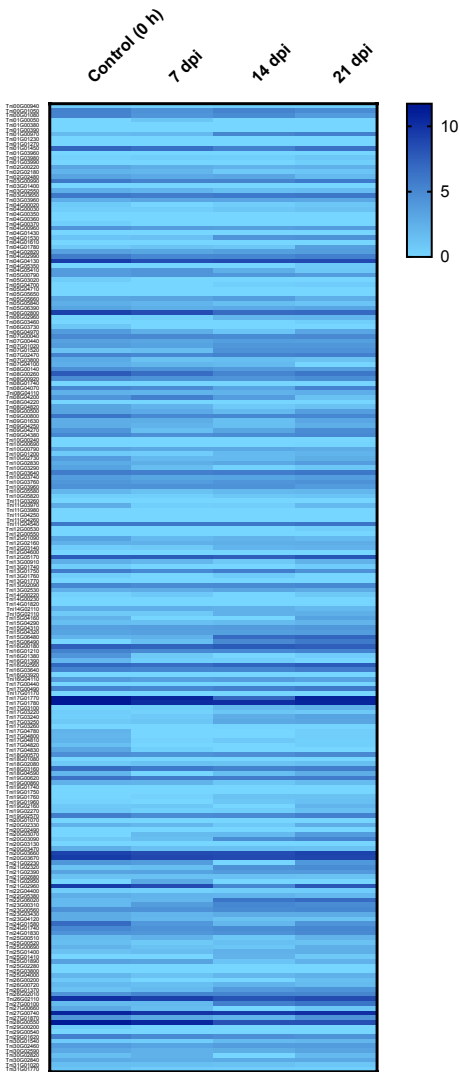

**D**

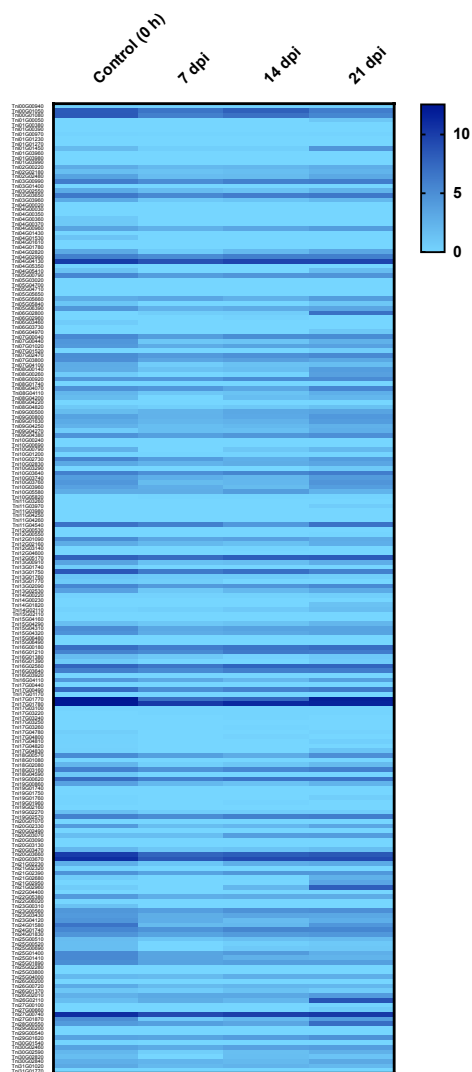

Supplement: Supplementary file 1 [file viruses-14-01444-s001.zip › Supplementary Table and figure/Supplementary Figure S1.pdf]
